# Potential roles of the endoplasmic reticulum stress pathway in amyotrophic lateral sclerosis

**DOI:** 10.3389/fnagi.2023.1047897

**Published:** 2023-02-15

**Authors:** Yu-Mi Jeon, Younghwi Kwon, Shinrye Lee, Hyung-Jun Kim

**Affiliations:** ^1^Dementia Research Group, Korea Brain Research Institute, Daegu, Republic of Korea; ^2^Department of Brain and Cognitive Sciences, Daegu Gyeongbuk Institute of Science and Technology (DGIST), Daegu, Republic of Korea

**Keywords:** amyotrophic lateral sclerosis, endoplasmic reticulum stress, unfolded protein response, therapeutic target, motor neuron disease

## Abstract

The endoplasmic reticulum (ER) is a major organelle involved in protein quality control and cellular homeostasis. ER stress results from structural and functional dysfunction of the organelle, along with the accumulation of misfolded proteins and changes in calcium homeostasis, it leads to ER stress response pathway such as unfolded protein response (UPR). Neurons are particularly sensitive to the accumulation of misfolded proteins. Thus, the ER stress is involved in neurodegenerative diseases such as Alzheimer’s disease, Parkinson’s disease, prion disease and motor neuron disease (MND). Recently, the complex involvement of ER stress pathways has been demonstrated in experimental models of amyotrophic lateral sclerosis (ALS)/MND using pharmacological and genetic manipulation of the unfolded protein response (UPR), an adaptive response to ER stress. Here, we aim to provide recent evidence demonstrating that the ER stress pathway is an essential pathological mechanism of ALS. In addition, we also provide therapeutic strategies that can help treat diseases by targeting the ER stress pathway.

## 1. Introduction

Motor neuron disease (MND) refers to a group of rare neurodegenerative diseases that result in a progressive loss of motor function due to selective degeneration of upper motor neurons and/or lower motor neurons (Gagliardi et al., 2021). The cell bodies of motor neurons are in the motor cortex, brainstem or spinal cord, and they control skeletal muscle activity, including breathing, speaking, walking, and swallowing. Motor neuron disease classified two ways: (1) familiar or sporadic, (2) symptomatically. In symptomatically, MND divided into upper motor neuron degeneration (UMN) and lower motor neuron degeneration (LMN; Statland et al., 2015). Motor neuron disease includes amyotrophic lateral sclerosis (ALS), progressive muscular atrophy (DeJesus-Hernandez et al., 2011), progressive lateral sclerosis (PLS), spinal muscular atrophy and Kennedy’s disease (Krivickas, 2003). ALS is the most common and fatal type of MND. The typical feature of ALS is a sudden loss of muscle control and paralysis. Moreover, the average life expectancy of ALS after symptom onset is 2–5 years (van Es et al., 2017; Zhao et al., 2022). More than 90% of ALS cases are sporadic, and 5–10% of ALS patients have a family history of ALS (Hou et al., 2016; Vajda et al., 2017). Sporadic ALS and Familiar ALS also differ in the age of onset. Sporadic ALS cases occurred 58–63, familiar ALS cases are 47–52 approximately (Kiernan et al., 2011). Recent advances in genome sequencing facilitate the identification of new genes associated with ALS, so at least 40 ALS-causing and related genes have been discovered. However, *C9orf72*, *SOD1*, *TARDBP*, and *FUS* account for half of familial ALS cases (Goutman et al., 2022). There are only 2 FDA-approved medications used to treat ALS, riluzole and edaravone. Riluzole is involved in glutamatergic transmission, and edaravone reduces reactive oxygen species production. The drugs have been shown to prolong life by only a few months or to slow the progression of symptoms, rather than being an actual cure (Jaiswal, 2019; Sadr et al., 2021). The main pathological feature of ALS is abnormal accumulation of misfolded or aggregated proteins in affected neurons and glia. The major roles of the endoplasmic reticulum (ER) are precise folding of newly synthesized proteins and degradation of unfolded or misfolded proteins. Thus, abnormal aggregation of misfolded proteins found in ALS can trigger a stress response called the ER stress pathway. Numerous lines of evidence indicate that ER stress plays an important role in ALS (Ilieva et al., 2007; Vijayalakshmi et al., 2011; Qiu et al., 2013; Atkin et al., 2017; Bugallo et al., 2020).

The ER is an intracellular organelle found in eukaryotic cells. The main functions of the ER are the folding of secreted proteins, participating in calcium signaling as one of the largest calcium stores, and posttranslational modification (Gething and Sambrook, 1992; Helenius et al., 1992; Berridge, 2002). Ca^2+^ is responsible for the transmission of neuronal depolarization and synaptic activity, and Ca^2+^ homeostasis and signaling are responsible for proper synaptic plasticity and survival. Thus, disruption of Ca^2+^ homeostasis affects neurotoxicity and neuronal autophagy and eventually leads to the progression of neurodegenerative disorders (Tong et al., 2018; Mozolewski et al., 2021). The accumulation of unfolded/misfolded proteins within the ER results in stress, which is mitigated by a conserved signaling mechanism known as the highly unfolded protein response (UPR; Moon et al., 2018; Kadowaki and Nishitoh, 2019). However, chronic, or overwhelming ER stress is known to trigger a series of signaling mechanisms that promote cell death and inflammation. Therefore, ER dysfunction and persistent UPR activation may form a central link in the pathogenesis of ALS. In this review, we aim to provide insight into the role and complex interactions of the ER stress pathway and neurodegeneration in ALS.

## 2. Overview of the endoplasmic reticulum stress pathway

Endoplasmic reticulum is large compartment in eukaryotic cell, and it plays critical role in protein synthesis and folding, Lipid biogenesis, and Ca^2+^ metabolism (Koch, 1990; Reid and Nicchitta, 2015). These functions are essential for cell. Excessive stress disrupts ER homeostasis, it can lead ER stress. There are several pathways to maintain ER homeostasis: unfolded protein response (UPR), ER-associated degradation (ERAD), and ER-phagy. ERAD recognizes misfolded protein in ER, misfolded proteins are removed by 26S proteasome (Hampton, 2002; Lemus and Goder, 2014; Hwang and Qi, 2018). ER-phagy is one of the autophagy pathways to remove damaged ER (Yang et al., 2021).

**Figure 1:**
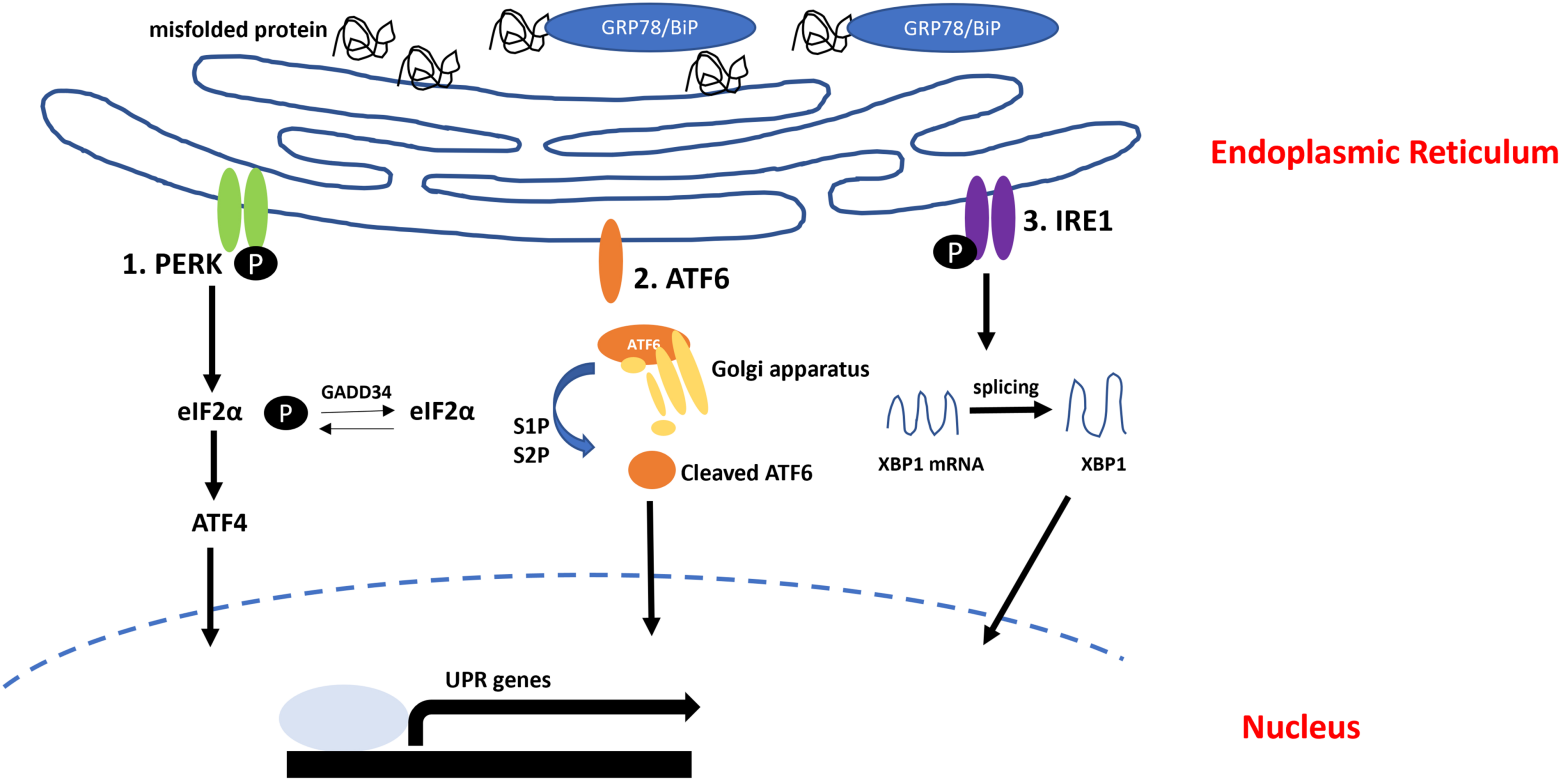
Unfolded protein response (UPR) pathway. In normal condition, GRP78/BiP interact with UPR sensors (PERK, ATF6, and IRE1). When misfolded or unfolded proteins accumulated in ER, GRP78/BiP dissociated from UPR sensors and can bind to misfolded or unfolded proteins. (1) Protein kinase RNA-like endoplasmic reticulum kinase (PERK) autophosphorylates and induces eIF2α phosphorylation. (2) Activating transcription Factor 6 (ATF6) moves into Golgi apparatus and cleaved by S1P and S2P. Cleaved ATF6 moves into nucleus, activates UPR genes. (3) IRE1 autophosphorylates and splices XBP1.

The accumulation of unfolded or misfolded proteins in the lumen of the ER induces ER stress, and this stress initiates an interrelated adaptive pathway known as the UPR to restore ER homeostasis. This pathway restores cellular function by halting protein translation, activating signaling pathways that degrade misfolded proteins and increasing the production of molecular chaperones (Mahdi et al., 2016; Nguyen and Uhal, 2016). In addition, activation of the UPR is associated with the generation of proinflammatory conditions (Janssens et al., 2014; Liu et al., 2017). In mammalian cells, the UPR signaling pathway is initiated by three ER membrane-associated sensors: inositol-requiring transmembrane kinase/endoribonuclease 1α (IRE1α), protein kinase RNA-like endoplasmic reticulum kinase (PERK) and activating transcription Factor 6 (ATF6). In normal condition, three sensors interact with 78 kDa glucose-regulated protein/Binding immunoglobulin protein (GRP78/BiP). GRP78/BiP is ER resident chaperon, it acts as a regulator or UPR signaling. Upon misfolded or unfolded protein accumulate in ER, GRP78/BiP dissociated from UPR sensors. After that, PREK and IRE1 form homo-dimerization, respectively, and autophosphorylate (Lee, 2005). Also, ATF6 is moved into Golgi apparatus and cleaved by S1P and S2P proteases. Cleaved ATF6 translocated into nucleus (Figure 1; Guzel et al., 2017).

The first branch of the UPR is IRE1, an ER transmembrane sensor. It is perfectly conserved in yeast and animals (Chen and Brandizzi, 2013; Ashford et al., 2021). IRE1 has two isoforms, α and β, and regulates many cellular processes involved in cell survival and apoptosis. Activation of IRE1 is triggered by dissociation of the major ER chaperone, GRP78/BiP, from the luminal domain of IRE1. This dissociation induces dimerization and trans-autophosphorylation, finally triggering the RNase domain activity of IRE1 (Nikawa and Yamashita, 1992; Pincus et al., 2010; Riaz et al., 2020). Eventually, X-box binding protein 1 (XBP1) mRNAs, which are processed by IRE1, translocate to the nucleus and are implicated in the transcriptional upregulation of ER chaperones or adaptive UPR factors for the degradation of misfolded proteins (Yoshida et al., 2001a; Kimata et al., 2004; Junjappa et al., 2018). However, overloaded IRE1 increases the translation of apoptosis-associated proteins by shutting down microRNA (anti-apoptotic) biosynthesis and mediates cell death activation (Upton et al., 2012; Demirel-Yalciner et al., 2021).

Protein kinase RNA-like endoplasmic reticulum kinase is another transducer of unfolded protein response. PERK relieves excessive ER stress by reducing global protein synthesis (Haze et al., 1999; Junjappa et al., 2018; Ohno, 2018). Activation of PERK is triggered by dimerization and autophosphorylation of PERK. Activated PERK shuts off global protein translation by preventing preinitiation complex formation in the ribosome through eukaryotic initiation Factor 2α (eIF2α) phosphorylation at serine 51 (Harding et al., 1999; Humeau et al., 2020). Phosphorylated eIF2α inhibits GDP-GTP exchange, reducing the level of the translationally functional ternary complex and thereby inhibiting translation initiation. However, prolonged attenuation of global protein synthesis leads to disruption of cellular protein homeostasis. Interestingly, PERK-mediated eIF2α phosphorylation has been associated with various diseases, including neurodegenerative disorders. In particular, phosphorylated PERK and phosphorylated eIF2α were significantly increased in postmortem brain tissue of patients with Alzheimer’s disease (AD; Chang et al., 2002; Hoozemans et al., 2005; Lourenco et al., 2015), and similar results were reported in several AD animal models (Devi and Ohno, 2010, 2014). In addition, other neurodegenerative diseases, such as prion disease (Moreno et al., 2012), ALS (Saxena et al., 2009), Parkinson’s disease (Colla et al., 2012) and Huntington’s disease (Leitman et al., 2014), have been associated with the activation of PERK.

The last branch of the UPR is mediated by ATF6. ATF6 is an ER-associated type II membrane-bound protein (Horimoto et al., 2013). When the unfolded proteins accumulate in the ER, ATF6 is translocated from the ER to the Golgi apparatus and is sequentially cleaved by site-specific Site-1 protease and Site-2 protease (Ye et al., 2000). Cleaved ATF6 is translocated to the nucleus to induce transcription of genes encoding ER chaperones and ERAD pathway component genes. In addition, ATF6 also plays a role in the transcriptional upregulation of apoptosis genes (Teske et al., 2011).

**Figure 2:**
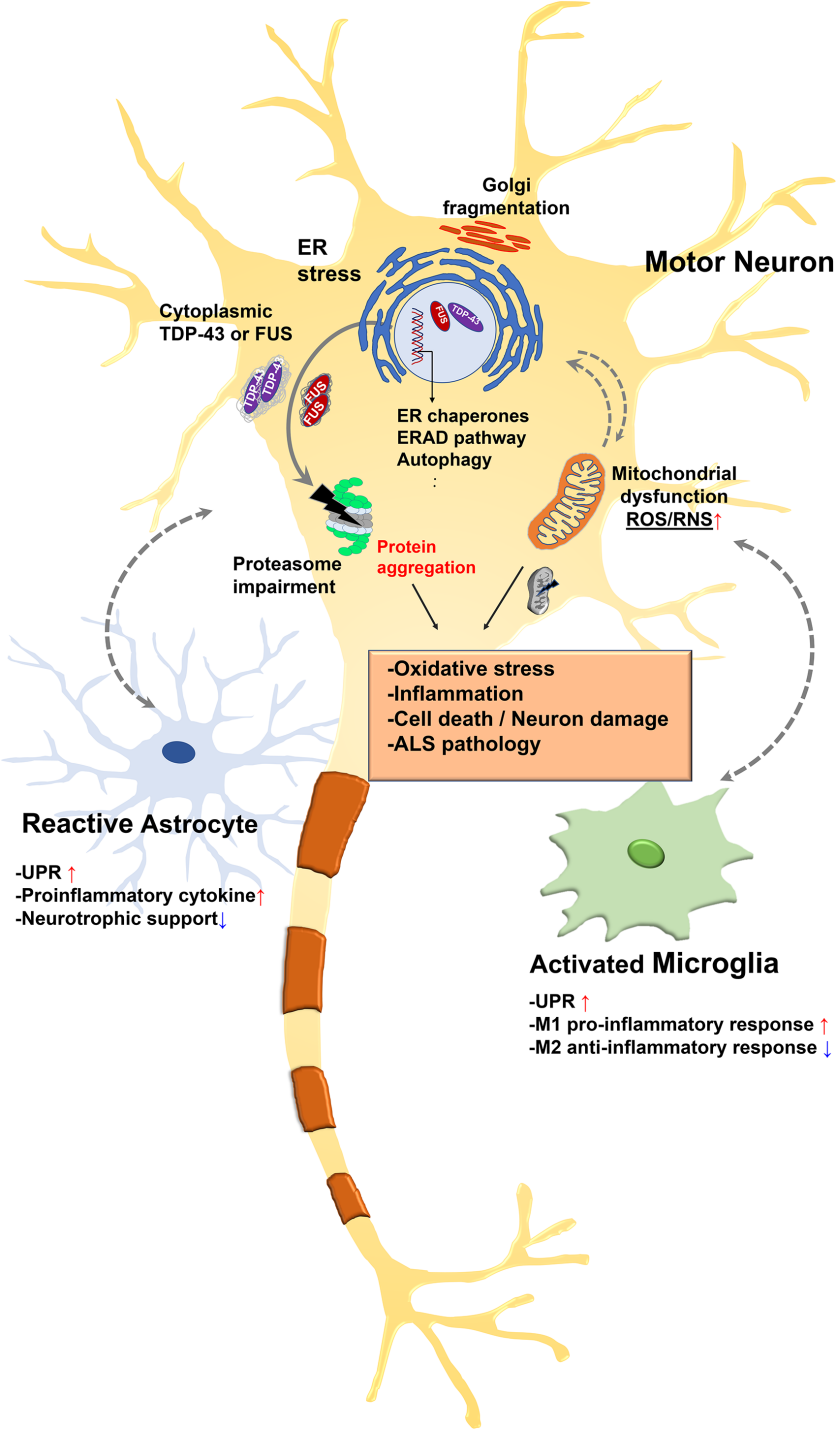
Relationship between endoplasmic reticulum (ER) stress and amyotrophic lateral sclerosis (ALS)-associated pathology. Misfolded proteins accumulate in the ER lumen and this accumulation contributes to ER stress. ER stress triggers the UPR to restore protein homeostasis. However, chronic stress leads to a cascade of intracellular death and inflammatory signal. Some nuclear ALS disease proteins such as TAR DNA-binding protein 43 (TDP-43) or fused in sarcoma (FUS) are depleted in the nucleus and accumulate in the cytoplasm in pathologic condition. Pathologic TDP-43 or FUS are existed as hyperphosphorylated and ubiquitinated form. Persistent activation of the stress response leads to ER dysfunction and ubiquitin proteasome system (UPS) impairment, leading to reactive oxygen species (ROS) and RNS release in mitochondria. This eventually leads to insoluble cytoplasmic aggregation and impairment of mitochondrial and ER function. Furthermore, UPR-related genes are upregulated in astrocytes and microglia along with inflammatory genes. Specifically, UPR activation reduces the ability of glia to support synapse and is associated with neuronal cell death. In addition, glia could transmit ER stress to neurons, which in turn may exacerbate ER stress mediated neuronal damage.

Chronic ER stress induces apoptotic cell death *via* the UPR. Thus, cell death caused by prolonged ER stress is increasingly recognized as a common cause of a wide range of diseases, including neurodegeneration (Moreno et al., 2012; Ismael et al., 2021), inflammation (Bellezza et al., 2014), metabolic disorders (Lemmer et al., 2021), cancer (Samanta et al., 2021), diabetes (Shih et al., 2021), and cardiovascular diseases (Ren et al., 2021). Persistent activation of UPR is associated with ALS and many other neurodegenerative diseases, and recent studies have shown that it is associated with neurons as well as non-neuronal cells such as astrocytes and microglia (Figure 2; Ito et al., 2009; Walker and Atkin, 2011; Sims et al., 2022). Therefore, inhibition of the UPR may be a promising therapeutic strategy for these diseases.

## 3. The relationship between ER stress and ALS-associated genes

### 3.1. Superoxide dismutase 1

Superoxide dismutase 1 (SOD1) is an antioxidant enzyme that protects against oxidative stress in eukaryotic cells (Trist et al., 2021). An association between SOD1 and familial ALS (fALS) was identified in 1993 (Rosen et al., 1993). ALS patients associated with *SOD1* mutation contain SOD1-immunoreactive inclusions (Bruijn et al., 1998). In fALS cases, up to 20% of cases have SOD1 mutations; to date, more than 180 mutations have been identified in ALS cases (Hayashi et al., 2016). SOD1^G93A^ mice were the first transgenic mice used as an ALS model, and their phenotype is very similar to that of ALS in humans (Gurney et al., 1994). The ALS-associated SOD1 mutation induces several pathological features, such as oxidative stress (Ferrante et al., 1997), mitochondrial dysfunction (Mattiazzi et al., 2002; Damiano et al., 2006), and prion-like propagation of disease proteins (Grad et al., 2014). Furthermore, ER stress is an important SOD1-related pathology (Nagata et al., 2007; Nishitoh et al., 2008). In SOD1^G93A^ mutant mice, increased phosphorylation of PERK and eIF2α was observed. Increased ER stress markers were found at the mid-to-late presymptomatic stage in SOD1^G93A^ transgenic mice. Following the response of PERK/eIF2α phosphorylation, Caspase12 expression is also increased. Caspase 12 is located on the outer surface of the ER, and its activation elicits the proapoptotic caspase cascade. Thus, these results indicate that ER stress leads to cell death in mutant SOD1 mice (Nagata et al., 2007).

Endoplasmic reticulum-associated degradation (ERAD) targets misfolded proteins in ER, misfolded proteins transported from ER to cytosol, where they are degraded by ubiquitin proteasome system (UPS) under activated UPR conditions. Inhibition of ERAD disrupts the cytosolic transport of misfolded proteins, and they accumulate in the ER and induce ER stress (Kopito, 1997; Tsai et al., 2002). Moreover, mutant SOD1 directly binds to Derlin-1 in the ER (Nishitoh et al., 2008) and that Derlin-1 is an important protein in ERAD-mediated translocation from the ER to the cytosol (Lilley and Ploegh, 2004; Ye et al., 2004). The interaction between the SOD1 mutant and Derlin-1 inhibits the translocation of misfolded proteins from the ER to the cytosol, and misfolded proteins accumulate in the ER. The mutant SOD1-Derlin1 interaction induces IRE1/ASK1 signaling and cell death (Nishitoh et al., 2008).

It is known that phosphorylation of PERK induces ATF4 expression through eIF2α phosphorylation. Consistently, SOD1 mutant ALS mice showed upregulated ATF4 expression compared to control mice (Kikuchi et al., 2006). Moreover, upregulated UPR genes were related to accumulation of SOD1 in the ER. Under normal conditions, BiP interacts with UPR sensing proteins such as PERK, ATF6, and IRE-1. In SOD1 mutant ALS mice, the binding affinity between BiP and other UPR sensors is increased (Kikuchi et al., 2006).

Protein disulfide isomerase A1 (PDIA1, refer to PDI) is a chaperone that localizes to the ER. ER stress upregulates PDI, and it plays a protective role in protein misfolding and aggregation (Parakh and Atkin, 2015). PDI interacts with misfolded proteins rather than native proteins in the ER through hydrophobic interactions (Parakh and Atkin, 2015). PDI promotes the translocation of misfolded proteins from the ER to the cytosol *via* ERAD, and misfolded proteins are degraded by the UPS (Molinari et al., 2002; Lee et al., 2010). Overexpression of PDI reduces mutant SOD1 aggregation in NSC-34 motor neuron like cell lines. Moreover, the expression of BiP, CHOP, and PERK phosphorylation was decreased by PDI overexpression in NSC-34 cells. Several studies have reported that ER stress in the SOD1 ALS model induces apoptotic cell death. PDI overexpression mitigates SOD1 mutant-induced cell death, and knockdown of PDI increased mutant SOD1 inclusions in NSC-34 cells (Walker et al., 2010). Based on this, ER stress-related chaperones might be mitigate mutant SOD1-induced ER stress.

### 3.2. Transactive response DNA-binding protein 43

Cytoplasmic aggregation of TAR DNA-binding protein 43 (TDP-43) is related to many neurodegenerative diseases referred to as TDP-43 proteinopathies (Kwong et al., 2007; Scotter et al., 2015). Cytoplasmic TDP-43 aggregate is a major pathologic hallmark of ALS (Neumann et al., 2006). More than 97% of ALS patients have TDP-43-positive inclusions in their affected neurons and glia (Mackenzie et al., 2007; Maekawa et al., 2009). Several studies have shown that overexpression of wild-type or ALS-associated mutant TDP-43 causes neurotoxicity (Ash et al., 2010; Kabashi et al., 2010; Li et al., 2010; Wils et al., 2010; Cascella et al., 2016). Immunoelectron microscopy data showed that TDP-43 was decreased in the nucleus and increased in the ER in ALS patients compared with controls (Sasaki et al., 2010). This research indicated that nuclear depletion of TDP-43 was related to TDP-43 accumulation in the ER (Sasaki et al., 2010; de Mena et al., 2021). Furthermore, overexpression of disease-associated TDP-43 mutations (D169G, G294A, A315T, Q331K, M337V, and N390D) increased cytoplasmic accumulation and induced UPR more than wild-type TDP-43 overexpression (Walker et al., 2013; Wang et al., 2015). Overexpression of TDP-43 also upregulates CHOP mRNA levels, and TDP-43-induced cell death is mediated by CHOP (Suzuki and Matsuoka, 2012). CHOP expression is regulated by the UPR such as PERK/eIF2α/ATF4 pathway. A previous study showed that TDP-43-induced cell death is also related to Bim activation (Suzuki et al., 2011) and that CHOP acts as a transcription factor for the Bim expression (Puthalakath et al., 2007). Moreover, CHOP expression is more strongly induced by mutant TDP-43 compared to wild type TDP-43 (Walker et al., 2013). These results suggest that TDP-43 accumulation in the ER can lead to ER stress and ER stress is implicated in TDP-43 induced neurotoxicity.

Many studies have shown that wildtype or mutant TDP-43 can increase eIF2α phosphorylation. Overexpression of TDP-43 in *Drosophila* nervous system increased the phosphorylation of eIF2α (Ser51; Kim et al., 2014). Moreover, increased eIF2α phosphorylation is essential in TDP-43-mediated neurotoxicity in *Drosophila*. Inhibition of PERK using GSK2606414 (PERK inhibitor) reduced eIF2α phosphorylation and rescued TDP-43-mediated neurotoxicity in *Drosophila* and mammalian neurons. Inhibition of GADD34 (eIF2α phosphatase) also enhances eIF2α phosphorylation and accelerates TDP-43-induced neurotoxicity (Kim et al., 2014). In addition, overexpression of TDP-43 in SH-SY5Y human neuroblastoma cells increased eIF2α phosphorylation, and increased eIF2α phosphorylation upregulates the transcription levels of ER stress-related genes such as *ATF4*, *CHOP*, and *GADD34*. Similarly, a reduction in eIF2α phosphorylation restores the transcription level of ER stress-related genes and mitigates TDP-43-induced cell death in SH-SY5Y cells (Jeon et al., 2021).

Protein kinase R (PKR or eukaryotic translation initiation factor-2 alpha kinase 2, EIF2AK2) regulates diverse cellular responses and is also associated with ER stress-related apoptosis through eIF2α phosphorylation, CHOP, and the ATF4 pathway. Under ER stress conditions, activation of PKR directly phosphorylates eIF2α in a PERK-independent manner and induces *CHOP* and *ATF4* expression (Lee et al., 2007). Previous study indicated that knockdown of TDP-43 in astrocytes induces a robust increase in eIF2α phosphorylation, which is attenuated by PKR inhibition (LaRocca et al., 2019). Furthermore, TDP-43^A315T^ and TDP-43^Q331K^ promote neuronal toxicity in SH-SY5Y cells *via* ER stress pathway, such as eIF2α phosphorylation, CHOP, and GRP78 (Wang et al., 2015; Hu et al., 2019). In ALS patients who have the TDP-43^A315T^ mutation, the ER stress-related chaperone GRP78 is increased compared to that of controls (Wang et al., 2015).

Activation of the IRE-1 mitigates ER stress mediated cytotoxicity *via* upregulation of *XBP1* splicing (Yoshida et al., 2001a; Dafinca et al., 2021). TDP-43^M337V^ expression in rat neurons decreased spliced XBP1. Moreover, ubiquitin aggregation and Golgi fragmentation were also observed in the neurons of TDP-43^M337V^ transgenic rats (Tong et al., 2012). On the other hand, another ALS-related mutant (TDP-43^Q331K^, TDP-43^A315T^) overexpression increased *XBP1* and *ATF6* expression compared with wild-type TDP-43 overexpression in N2a neuronal cells (Walker et al., 2013). Although relationship between XBP1 and TDP-43 mutant is controversial, TDP-43-related ER stress is associated with IRE1/XBP1 signaling pathways.

### 3.3. Fused in sarcoma

Fused in sarcoma (Li et al., 2010) was initially identified as a transcriptional regulator in human malignant liposarcomas in 1993 (Rabbitts et al., 1993). In 2009, a FUS mutation was identified in ALS patients (Kwiatkowski et al., 2009; Vance et al., 2009). In ALS patients, ~ 4% of FUS mutations are found in fALS, ~ 1% of mutations are found in sporadic ALS cases, and to date, more than 50 FUS mutations have been shown in ALS patients (Zou et al., 2017; Kim et al., 2020). FUS aggregation were observed in the spinal cord of ALS patients with *FUS* mutation, and FUS inclusion was colocalized with TDP-43 and ubiquitin (Deng et al., 2010). FUS is a DNA/RNA-binding protein; similar to TDP-43. FUS predominantly localizes to the nucleus (Lattante et al., 2013) and can shuttle between the nucleus and cytoplasm (Zinszner et al., 1997). Disease associated FUS mutations facilitate the cytoplasmic mislocalization of FUS. FUS^P525L^ and FUS^R522G^ mutants impair transportin mediated nuclear transport of FUS (Dormann et al., 2010).

Amyotrophic lateral sclerosis (ALS) linked mutant FUS induce ER stress, and overexpression of mutant FUS colocalized with calreticulin (as a marker of ER) in NSC-34 motor neuron cells. Overexpression of mutant FUS increases the overlap with calreticulin compared to wildtype FUS overexpression, and cytoplasmic mislocalization of FUS is also increased compare with wildtype FUS in NSC-34 (Farg et al., 2012). Moreover, mutant FUS expression induces *XBP1* splicing and *CHOP* expression (Farg et al., 2012). These results indicated that cytoplasmic aggregation of FUS correlates with ER stress.

Fused in sarcoma (FUS) is also related to PDI. Overexpression of the FUS mutant in NSC-34 cells increased PDI expression compared to wildtype FUS overexpression. ALS patients who have the FUS^R521C^ mutation showed colocalization of FUS with PDI in the spinal cord (Farg et al., 2012). A recent study showed that overexpression of PDI reduces mutant FUS-induced ER stress. Overexpression of mutant FUS induces nuclear translocation of CHOP. However, overexpression of PDI decreases the nuclear immunoreactivity of CHOP in mutant FUS-overexpressing cells (Parakh et al., 2021). FUS is a RNA-binding protein with a prion-like domain that can form aggregates (Gitler and Shorter, 2011). A previous study showed that PDI can interact with prion-like proteins, suggesting that PDI can bind FUS through prion-like domains (Watts et al., 2009). Thus, enhancing the interaction between FUS and PDI might be the promising therapeutic strategy for FUS mediated neurodegeneration.

### 3.4. Chromosome 9 open reading frame 72

The expansion of the GGGGCC (G_4_C_2_) hexanucleotide in the chromosome 9 open reading frame 72 (C9orf72) gene accounts for 10% of ALS patients. Among them, ~ 40% are fALS cases (DeJesus-Hernandez et al., 2011; Yang et al., 2020), and 5 ~ 10% are sporadic cases (Umoh et al., 2016). There are several pathological features in C9orf72-related diseases, including toxic gain or loss of function in C9orf72, dipeptide repeat (DPR) proteins generated by repeat-associated non-ATG (RAN) translation, and transcriptional silencing (Dafinca et al., 2021). It is known that RAN translation of the C9orf72 gene produces five DPRs, glycine–alanine (GA), glycine-proline (Kim et al., 2022), glycine-arginine (GR), proline-alanine (PA), and proline-arginine (PR; Freibaum and Taylor, 2017).

Transcriptome analysis in the cerebellum and frontal cortex of C9orf72-ALS patients revealed altered UPR-related gene expression such as *ATF4* and *CHOP* (Freibaum and Taylor, 2017). Phosphorylation of PERK, another marker for ER stress, was also increased in the brain of C9orf72-ALS patient. Expression of poly-GA proteins induces neuronal toxicity through ER stress in mouse primary cortical neurons (Zhang et al., 2014). Overexpression of poly-PR using lentivirus in primary cortical neurons showed that ER stress-related genes were highly upregulated by PR, and PR expressing cells also showed increased neuronal toxicity. Moreover, the mRNA levels of *ATF4*, *CHOP*, and *GADD34* were also upregulated by poly-PR expression in K562 cell lines (Kramer et al., 2018). Consistent with this research, treatment of SH-SY5Y cells and primary cortical neurons with the poly-PR peptide increased the neuronal toxicity and transcription levels of *ATF4* and *CHOP* in a dose-dependent manner (Wang et al., 2019). Interestingly, recent research has revealed a relationship between RAN translation of C9orf72 and eIF2α phosphorylation (Cheng et al., 2018). Repeat-associated non-ATG (RAN) translation of C9ORF72 hexanucleotide repeats are increased by excessive phosphorylation of eIF2α. All these data indicated that ER stress is related to C9ORF72-mediated neurodegeneration.

### 3.5. Others

#### 3.5.1. Optineurin

Optineurin (OPTN) is associated with several cellular functions, such as the inflammatory response, autophagy, and Golgi maintenance (Toth and Atkin, 2018). More than 20 mutations in OPTN are associated with ALS. Unlike other ALS-causing mutation, TDP-43, SOD1, and FUS, OPTN mutations do not facilitate protein aggregation (Markovinovic et al., 2017). Overexpression of the ALS-associated OPTN^E478G^ mutation increased the annexin V/7-AAD-positive cells in NSC-34, indicating that mutant OPTN induces cell death (Sayyad et al., 2017). A 2-base pair insertion in *OPTN* (619-692insAG or 2 bpIsn-OPTN) is associated with both ALS and glaucoma. 2 bpIsn-OPTN increased caspase-3 activation and ER stress in NSC-34 cells. Overexpression of 2 bpIsn-OPTN in NSC-34 cells upregulated the transcription of *CHOP* and *XBP1*. Moreover, treatment with an ER stress inhibitor decreased ER stress-related gene expression and reduced cell death. 2 bpIsn-OPTN is predominantly localized in the nucleus, in contrast to wild-type OPTN mainly localized in the cytoplasm (Medchalmi et al., 2021). Loss of OPTN in mouse and MEF cells also induced upregulation of ER stress pathway (Ramachandran et al., 2021). OPTN knockout cells are more susceptible to cytotoxicity of ER stress inducers (tunicamycin and thapsigargin), and UPR-related genes were also highly upregulated. Moreover, basal levels of PERK and IRE1 were significantly higher in OPTN knockout MEFs. OPTN deletion also increased the mRNA level of *CHOP* in the tunicamycin treated mouse brain (Ramachandran et al., 2021). These results suggest that loss of OPTN can induce cell death through ER stress pathway.

#### 3.5.2. Ubiquitin-like protein ubiquilin2

The ubiquitin-like protein ubiquilin2 (UBQLN2) mutation is related to fALS cases, but the pathological features of UBQLN2 are largely unknown. However, UBQNL2 is involved in ERAD mediated protein degradation *via* UPS. Moreover, the best described UBQLN2-associated pathologies are the UPS and Autophagy impairment (Renaud et al., 2019). The viral expression of ALS linked UBQLN2 mutations (P497H, P497S, and P506T) in mouse brain caused reduction of motor capacity and accumulation of UBQLN2 containing inclusions (Ceballos-Diaz et al., 2015). Moreover, UBQLN2^P497H^ and UBQLN2^P506T^ overexpression in Neuro2A cells upregulates ER stress mediators such as XBP1 and CHOP compared to wildtype UBQLN2 (Halloran et al., 2020). Taken together, these data indicate that ER stress might be implicated in UBQLN2 induced neurodegeneration.

## 4. Endoplasmic reticulum stress as a therapeutic target for ALS

### 4.1. Salubrinal

Salubrinal is selective inhibitor of eIF2α phosphatase (Lu et al., 2022). Salubrinal upregulates eIF2α phosphorylation and reduces ER stress-induced cytotoxicity in PC12 cells (Boyce et al., 2005). In SOD1^G93A-^ and SOD1^G85R^-transfected N2a cells, salubrinal treatment prevented SOD1-induced cell death through regulation of eIF2α phosphorylation (Oh et al., 2008). Furthermore, salubrinal treatment reduced TDP-43-induced neurotoxicity in *C. elegans* and zebrafish. Mutant TDP-43-expressing *C. elegans* and zebrafish showed motor neuron defects, and salubrinal treatment attenuated TDP-43 induced motor neuron degeneration (Vaccaro et al., 2013). Salubrinal-treated SOD1^G93A^ mice showed alleviation of disease progression, such as increased survival ability and reinnervation of neuromuscular junctions (NMJs; Saxena et al., 2009).

### 4.2. Arimoclomol

Arimoclomol, called BRX-220, is a co-inducer of heat-shock protein (Hargitai et al., 2003) and has been shown to be a therapeutic candidate in several diseases (Fog et al., 2018). Several studies have suggested that treatment with arimoclomol in ALS models prevents disease-associated pathology. Arimoclomol-treated SOD1^G93A^ mice had an increased lifespan (Kieran et al., 2004), and the delayed overall disease progression (Kalmar et al., 2008). The heat shock response (HSR) is essential for recovery of proteotoxic damage and associated with refolding and degradation of misfolded or unfolded proteins (Westerheide and Morimoto, 2005). Arimoclomol treatment restored HSP70 expression which is decreased by mutant SOD1 and prevented ER stress-induced motor neuron death in SOD1^G93A^ mice (Kieran et al., 2004; Kalmar et al., 2008). However, phase III clinical trial for arimoclomol against ALS failed in 2021. The ORARIALS-01 phase 3 trial (Clinicaltrialsgov identifier: NCT03491462) investigated 245 adults with ALS across North America and Europe receiving arimoclomol or placebo once daily for 76 weeks. As a result of evaluating the effect of arimoclomol through an integrated evaluation of function and survival, it was announced that the goal was not achieved^1^.

### 4.3. PRE-084

PRE-084 is a sigma-1 receptor agonist. The sigma-1 receptor is a transmembrane receptor that localizes to the ER and mitochondria-associated ER membrane (MEM; Mancuso and Navarro, 2017). It has neuroprotective effects that regulate Ca^2+^ signaling, mitochondrial function, and ER stress (Hayashi and Su, 2007; Sharma et al., 2021). The sigma-1 receptor can translocate between the ER and MAM junction and plays a critical role in ER-mitochondria communication. The sigma-1 receptor directly binds to Bip, and it respond to ER stress and regulate Ca^2+^ homeostasis (Hayashi and Su, 2007). Several studies have shown that sigma-1 receptor is associated with ALS pathology. In SOD1^G93A^ mice, deletion of the sigma-1 receptor accelerated motor neuron dysfunction and decreased longevity (Mavlyutov et al., 2013). Another study showed that loss of the sigma-1 receptor affected the mitochondria-ER connection, leading to ER stress and dysfunction of Ca^2+^ signaling in culture mouse motor neurons (Bernard-Marissal et al., 2015). Furthermore, mutation of the sigma-1 receptor was found in ALS patients, and this mutation induced neuronal cell death through ER stress in NSC-34 motor neuron-like cells (Al-Saif et al., 2011; Bernard-Marissal et al., 2015). PRE-084 treatment in SOD1^G93A^ mice rescued motor neuron dysfunction, including survival, through PCK activation (Mancuso et al., 2012). SA4503, another sigma-1 receptor agonist, also reduced SOD1^G93A^-induced cell death and extended the survival time of SOD1^G93A^ mice (Ono et al., 2014).

### 4.4. Sephin1

Regulators of the PERK pathway (Salubrinal, Sephin1 and Guanabenz) inhibit the protein phosphatase complex to prevent dephosphorylation of eIF2α, a negative feedback loop that regulates protein translation during ER stress. Selphin1 is a selective inhibitor of GADD34 (PPP1R15A), a stress-induced regulatory subunit of the protein phosphatase 1 complex that dephosphorylates eIF2α (Das et al., 2015; Chen et al., 2021). Sephin1 has shown a strong effect in preventing motor neuron degeneration in *in vitro* and *in vivo* ALS models (Das et al., 2015; Chen et al., 2019). Sephin1 prevented the accumulation of the insoluble SOD1 and decreased the ER stress markers including Bip, CHOP, and Xbp-1 in spinal cords of SOD1^G93A^ mice. Thus, Sephin1 mitigated motor deficits, motor neuron loss, and molecular defects in SOD1 mutant mice (Das et al., 2015).

### 4.5. Guanabenz

Guanabenz is an FDA-approved alpha-2 adrenergic receptor agonist used as an antihypertensive agent. Guanabenz has been shown to prevent the accumulation of misfolded proteins and ER overload by regulating protein synthesis (Tsaytler et al., 2011). An *in vitro* study showed that Guanabenz prolonged eIF2α phosphorylation and modulated the rate of protein production in stressed human cells. This provided strong evidence that restoring protein homeostasis in stressed cells could be a drug target for ALS (Tsaytler et al., 2011). According to an *in vivo* study using the SOD1 mutant transgenic mice, Guanabenz attenuated ER stress due to prolonged eIF2α phosphorylation or downregulated the expression of proapoptotic proteins, thereby delaying disease onset, extending lifespan, and reducing motor neuron loss in mice (Jiang et al., 2014; Wang et al., 2014). Moreover, a phase II randomized clinical trial in ALS patients showed that a combination of guanabenz with riluzole slowed disease progression in early ALS patients with bulbar onset (Dalla Bella et al., 2021). As described above, many drugs that directly target UPR are summarized in a table (Table 1).

**Table 1:** Small molecules targeting the unfolded protein response (UPR) pathway for amyotrophic lateral sclerosis (ALS) treatment.

Small molecule	Molecular target	Model	Experimental evidence	Stage	References
Salubrinal	Block eIF2α dephosphorylation	SOD1^G93A^ mice	Alleviation of disease manifestations and delayed progression	Preclinical	Saxena et al. (2009)
		*Caenorhabditis elegans*/zebrafish expressing TDP-43^G348C^	Response to neurotoxicity through reduction of ER stress. Rescue of the motor phenotype		Vaccaro et al. (2013)
		Neuro2a cells expressing SOD1^G85R^/SOD1^G93A^	Protects cells by suppressing the UPR that propagates as a death signal		Oh et al. (2008)
Arimoclomol	Coinducer of heat shock proteins	SOD1^G93A^ mice	Improvement of muscle function and motor neuron(MN) survival	Fails phase III clinical trial	Kieran et al. (2004)
					Kalmar et al. (2008)
		ALS patients	Phase III clinical trial for 245 patients with ALS		https://clinicaltrials.gov/ct2/show/NCT03491462
PRE-084	Sigma-1 receptor agonist	SOD1^G93A^ mice	Improvement of MN function	Preclinical	Mancuso et al. (2012)
			Functional preservation of MNs and an increase in the number of surviving MNs		Gaja-Capdevila et al. (2021)
		Zebrafish expressing TDP-43^G348C^	Restoration of motor performance and mitochondrial respiration		Lasbleiz et al. (2022)
Sephin1	Selective inhibitor of GADD34	SOD1^G93A^ mice	Prevents motor deficits, motor neuron loss and molecular defects	*Phase II clinical trial*	Das et al. (2015)
		ALS patients	Treatment combining Riluzole and IFB-088 (sephin1) in bulbar-onset ALS		https://clinicaltrials.gov/ct2/show/NCT055080742
Guanabenz	Selective inhibitor of GADD34	SOD1^G93A^ mice	Attenuation of ER stress and mitochondrial stress. Delayed disease onset and extended lifespan in SOD1 ^G93A^ mice.	*Phase II clinical trial*	Jiang et al. (2014)
					Wang et al. (2014)
		*Caenorhabditis elegans*/zebrafish expressing TDP-43^G348C^	Response to neurotoxicity through reduction of ER stress.		Vaccaro et al. (2013)
			Rescue of the motor phenotype		
		ALS patients	200 patients within 18 months of onset of symptoms		Dalla Bella et al. (2021)
GSK2606414	Inhibit kinase domain of PERK	TDP-43 expressing *Drosophila*	Dramatic mitigation of TDP-43-induced climbing dysfunction	*In vivo* study	Kim et al. (2014)
		C9ORF72 expressing *Drosophila*	Inhibition of neurodegeneration in Drosophila		Zhang et al. (2018)
Cyclopiazonic acid	Specific inhibitor of SERCA	Motor neuron cultures from SOD1^G93A^ mice	Helps maintain cellular ER homeostasis by suppressing ER calcium uptake and inducing UPR	*In vitro* study	Lautenschlager et al. (2013)

## 5. Conclusion

Growing evidence indicates that the ER stress pathway is strongly implicated in the pathogenesis of ALS. Although the UPR is a cellular adaptive and protective response to overwhelming ER stress, sustained stress can result in an imbalance in protein homeostasis, resulting in increased ROS production. The UPR pathway is facilitated by the IRE1, PERK and ATF6 branches, which are important for maintaining protein homeostasis, and this protective role is becoming more important in ALS/MND. It is important to note that the UPR pathway may be affected equally or differently for each cell type involved. For example, microglia adopt an M2-like neuroprotective state early in disease but are likely to transition to an M1-like toxic state as ALS progresses (Liao et al., 2012; Chiu et al., 2013; Clarke and Patani, 2020).Therefore, a clearer and improved understanding of the relevant mechanisms of genetic and protein homeostatic dysfunction associated with the more complex ALS/MND will be essential and important for developing small molecule therapeutics that effectively target the UPR mediated ALS pathogenesis.

## Author contributions

Y-MJ, YK, and SL provided ideas for the project and participated in data collection. Y-MJ, YK, and H-JK wrote the paper. All authors contributed to the article and approved the submitted version.

## Funding

This work was supported by the KBRI Research Program of the Ministry of Science, ICT and Future Planning (22-BR-02-04; 22-BR-03-02) and the National Research Foundation of Korea (NRF) grant funded by the Korean government (MSIT; 2020R1A2C4002366; 2021R1C1C1008688).

## Conflict of interest

The authors declare that the research was conducted in the absence of any commercial or financial relationships that could be construed as a potential conflict of interest.

## Publisher’s note

All claims expressed in this article are solely those of the authors and do not necessarily represent those of their affiliated organizations, or those of the publisher, the editors and the reviewers. Any product that may be evaluated in this article, or claim that may be made by its manufacturer, is not guaranteed or endorsed by the publisher.

## Footnotes

^1^ https://clinicaltrials.gov/ct2/show/NCT03491462
